# Effects of hydrogen-rich water on depressive-like behavior in mice

**DOI:** 10.1038/srep23742

**Published:** 2016-03-30

**Authors:** Yi Zhang, Wen-Jun Su, Ying Chen, Teng-Yun Wu, Hong Gong, Xiao-Liang Shen, Yun-Xia Wang, Xue-Jun Sun, Chun-Lei Jiang

**Affiliations:** 1 Laboratory of Stress Medicine, Faculty of Psychology and Mental Health, Second Military Medical University, 800 Xiangyin Road, Shanghai 200433, China; 2 Department of Medicinal Chemistry, School of Pharmacy, Second Military Medical University, 325 Guohe Road, Shanghai 200433, China; 3 Department of Naval Aviation Medicine, Second Military Medical University, 800 Xiangyin Road, Shanghai 200433, China

## Abstract

Emerging evidence suggests that neuroinflammation and oxidative stress may be major contributors to major depressive disorder (MDD). Patients or animal models of depression show significant increase of proinflammatory cytokine interleukin-1β (IL-1β) and oxidative stress biomarkers in the periphery or central nervous system (CNS). Recent studies show that hydrogen selectively reduces cytotoxic oxygen radicals, and hydrogen-rich saline potentially suppresses the production of several proinflammatory mediators. Since current depression medications are accompanied by a wide spectrum of side effects, novel preventative or therapeutic measures with fewer side effects might have a promising future. We investigated the effects of drinking hydrogen-rich water on the depressive-like behavior in mice and its underlying mechanisms. Our study show that hydrogen-rich water treatment prevents chronic unpredictable mild stress (CUMS) induced depressive-like behavior. CUMS induced elevation in IL-1β protein levels in the hippocampus, and the cortex was significantly attenuated after 4 weeks of feeding the mice hydrogen-rich water. Over-expression of caspase-1 (the IL-1β converting enzyme) and excessive reactive oxygen species (ROS) production in the hippocampus and prefrontal cortex (PFC) was successfully suppressed by hydrogen-rich water treatment. Our data suggest that the beneficial effects of hydrogen-rich water on depressive-like behavior may be mediated by suppression of the inflammasome activation resulting in attenuated protein IL-1β and ROS production.

Major depressive disorder (MDD) is a pervasive mental disorder that affects about 350 million people across the world^1^. MDD ranks as the 11^th^ leading cause of disability-adjusted life years (DALYs), according to Global Burden of Disease studies measuring disease burden worldwide^2^. Decades of extensive studies in the pathophysiology of MDD have led to various hypotheses for the molecular basis of depression. Patients suffering from depression often display evidence of an inflammation characterized by the increased expression of proinflammatory cytokines such as interleukin-1 (IL-1), interleukin-6 (IL-6) and tumor necrosis factor (TNF)^3^. Proinflammatory cytokines can elicit sickness behavior and induce depressive-like neuroendocrine and central neurotransmitter changes which can be exacerbated by stressors^4^. Together, evidence from clinical and animal studies have led to the hypothesis that chronic cytokines expression may cause depressive illness in human beings^5^.

Cytokines in the periphery access the brain partly via afferent vagus nerve to elicit a sickness response in the central nervous system^6^. Peripheral nerve injury contributes to the development of depressive-like behavior via increasing cytokine expression. Stress facilitates this process by promoting the up-regulation of inflammatory cytokines such as IL-1β gene expression in the brain^7^. As a major regulator of stress responses, IL-1β can promote coping at low levels while exerting detrimental effects at high levels^8^. Studies by Goshen and colleagues demonstrate that elevation of brain IL-1 levels is necessary and sufficient for the development of depression^9^.

The expression and activation of the proinflammatory cytokine IL-1β is dependent on the assembly and activation of an intracellular protein complex called the inflammasome. Upon cellular infection or stress, the inflammasome complex mediate activation of Caspase-1 to promote the maturation of pro-IL-1β to its fully functional IL-1β form^10^. Interestingly, inflammasome dependent neuroinflammation pathways have recently been implicated in several CNS psychiatric illnesses^11^. Recent reports and reviews link inflammasome activation with major depressive disorder (MDD), and suggest that inflammasome activation may play a central role in the development of depressive-like behaviors^12^. Moreover, the NLRP3 inflammasome has been identified as a potential therapeutic target for alleviating neuroinflammation and for treatment of MDD^11^,^13^.

Oxidative stress may be a crucial contributor to the development of MDD. Patients with MDD show increased expression of inflammatory and oxidative stress biomarkers^14^. Oxidative stress results from a breakdown in homeostasis between reactive oxygen species (ROS) and antioxidants. Antioxidants can protect against ROS-induced neuronal damage by scavenging radicals and suppressing the oxidative stress pathway in the brain. Notably, Xu and colleagues have recently proposed antioxidants as a candidate treatment for depression, based on the reviews of recent studies on oxidative stress markers in patients and animal models of depression^15^.

Hydrogen selectively reduces cytotoxic oxygen radicals, and may potentially serve as a novel antioxidant in preventive and therapeutic applications^16^. Hydrogen-saline has been shown to be more convenient and efficient in protecting against inflammation than inhaling hydrogen gas^17^. Zhang and colleagues has reported that pretreatment with hydrogen-rich water could mitigate aspirin-induced gastric lesions through the inhibition of the oxidative stress and inflammatory reactions^18^. Hydrogen-rich saline suppresses the production of several proinflammatory mediators by inhibiting the activation of p38 and NF-κB. Furthermore, the therapeutic effects of hydrogen-rich saline in acute lung injury may be due to its antioxidant and anti-inflammatory actions^19^. Tomofuji and colleagues proposed that drinking hydrogen-rich water had anti-oxidative damage effects on aging periodontal tissues^20^.

Classic antidepressants, which are effective in less than 50% of patients, are often associated with a wide range of undesired side effects^21^. Therefore, identifying effective anti-depressive compounds with little to no side effects may serve as effective alternative or preventative treatments. It is unclear if hydrogen-rich water, which has no known side effects, may have beneficial effects in mitigating depression symptoms. We therefore investigated the preventative effects of hydrogen-rich water on the development of depressive-like behavior in the chronic unpredictable mild stress (CUMS) mouse model. We measured protein levels of IL-1β, detected caspase-1 activation and ROS production in the hippocampus and prefrontal cortex (PFC) to determine if hydrogen-rich water may exert its potential beneficial effects on depressive-like behavior. Our results demonstrate that hydrogen-rich water attenuates stress induced oxidative stress and neuroinflammation, in turn, preventing the development and progression of depressive-like behaviors following chronic stress exposure.

## Results

### Body weight before and after CUMS

**Figure 1:**
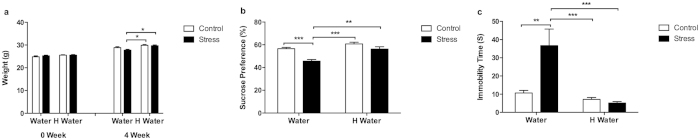
Body weight and depressive-like behavior. (**a**) After 4 weeks-long chronic unpredictable mild stress treatment, the stress group mice had significantly lower body weight when compared to the hydrogen-rich water and hydrogen-rich water + stress groups (F_1, 38_ = 15.74, P = 0.0003, post hoc *P < 0.01, n = 11, 11, 10, 10). (**b**) The stress group mice showed significantly lower sucrose preference percentage in sucrose preference test when compared to the control group (F_1, 38_ = 25.51, P < 0.0001, post hoc ***P < 0.0001, n = 11, 11, 10, 10), and compared to the hydrogen-rich water and hydrogen-rich water + stress groups (F_1, 38_ = 23.29, P < 0.0001, post hoc ***P < 0.0001, **P < 0.001, n = 11, 11, 10, 10). (**c**) The stress group mice showed significantly longer immobility time in tail suspension test when compared to the control group (F_1, 33_ = 8.784, P = 0.0056, post hoc **P < 0.001, n = 10, 8, 10, 9), and compared to the hydrogen-rich water and hydrogen-rich water + stress groups (F_1, 33_ = 18.64, P = 0.0001, post hoc ***P < 0.0001, n = 10, 8, 10, 9). No significant differences observed among the control, hydrogen-rich water and hydrogen-rich water + stress groups. Data was analyzed using a two-way ANOVA followed by the Bonferroni’s multiple comparisons post hoc test. Error bars represent s.e.m.

The baseline body weight of control group, hydrogen-rich water group, stress group and hydrogen-rich water + stress group had no significant differences. However, after 4 weeks of undergoing the CUMS procedure, the stress group had lower body weight compared to the control, hydrogen-rich water (p < 0.01) and hydrogen-rich water + stress groups (p < 0.01) (Fig. 1a).

### Depressive-like behavior

We performed the sucrose preference test and the tail suspension test to determine if the mice exhibited depressive-like behavior. The stress group mice exhibited a significantly lower sucrose preference percentage in the sucrose preference test (p < 0.001) (Fig. 1b), and significantly longer immobility times in the tail suspension test (p < 0.001) (Fig. 1c). The hydrogen-rich water ameliorated the stress-induced depressive-like behaviors as shown in the comparison between the hydrogen-rich water + stress group and stress group (Fig. 1b,c). There were no significant differences among control, hydrogen-rich water and hydrogen-rich water + stress groups.

### IL-1β levels in hippocampus and cortex

**Figure 2:**
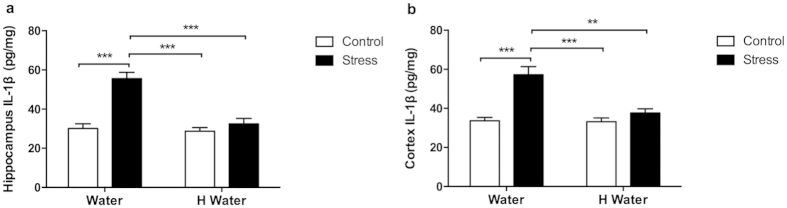
IL-1β level in hippocampus and cortex. (**a**) IL-1β levels in hippocampi of stress group mice increased significantly when compared to the control group (F_1, 16_ = 31.12, P < 0.0001, post hoc ***P < 0.0001, n = 5, 5, 5, 5), and compared to the hydrogen-rich water group (F_1, 16_ = 21.88, P = 0.0003, post hoc ***P < 0.0001, n = 5, 5, 5, 5). The hydrogen-rich water hindered the stress-induced increasing of IL-1β levels in hippocampi as shown in the comparison between hydrogen-rich water + stress group and stress group (F_1, 16_ = 21.88, P = 0.0003, post hoc ***P < 0.0001, n = 5, 5, 5, 5). (**b**) IL-1β levels in cortexes of stress group mice increased significantly when compared to the control group (F_1, 16_ = 27.70, P < 0.0001, post hoc ***P < 0.0001, n = 5, 5, 5, 5), and compared to the hydrogen-rich water group (F_1, 16_ = 14.27, P = 0.0016, post hoc ***P < 0.0001, **P < 0.001, n = 5, 5, 5, 5). The hydrogen-rich water inhibited the stress-induced increasing of IL-1β levels in cortexes as shown in the comparison between hydrogen-rich water + stress group and stress group (F_1, 16_ = 14.27, P = 0.0016, post hoc ***P < 0.0001, **P < 0.001, n = 5, 5, 5, 5). No significant differences observed among the control, hydrogen-rich water and hydrogen-rich water + stress groups. Data was analyzed using a two-way ANOVA followed by the Bonferroni’s multiple comparisons post hoc test. Error bars represent s.e.m.

The hippocampus IL-1β level of the stress group increased significantly compared to the control (p < 0.0001), hydrogen-rich water (p < 0.0001) and hydrogen-rich water + stress groups (p < 0.0001) (Fig. 2a). There were no significant differences among the latter three groups. The cortex IL-1β level of the stress group increased significantly as well compared to the other three groups (p < 0.001) (Fig. 2b). The hydrogen-rich water successfully mitigated the stress-induced elevation of IL-1β levels, as shown in the comparison between the hydrogen-rich water + stress group (p < 0.001) and stress group (Fig. 2a,b).

### Caspase-1 activity in hippocampus and prefrontal cortex

**Figure 3:**
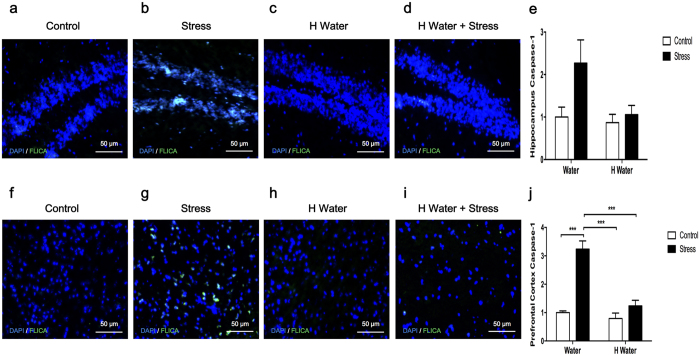
Caspase-1 activity in hippocampus and prefrontal cortex. ImmunoChemistry Technologies’ FAM-FLICA™ Caspase-1 Assay Kit was used to detect active caspase-1. The caspase-1 activity was expressed as relative of the control group. (**e**) Caspase-1 activity in hippocampi of the stress group mice increased when compared to the control, hydrogen-rich water and hydrogen-rich water + stress groups (F_1, 16_ = 4.814, P = 0.0433, n = 5, 5, 5, 5), which indicated activation of inflammasome. (**j**) Caspase-1 activity in prefrontal cortexes of the stress group mice increased significantly when compared to the control group (F_1, 16_ = 44.50, P < 0.0001, post hoc ***P < 0.0001, n = 5, 5, 5, 5), and compared to the hydrogen-rich water (F_1, 16_ = 30.06, P < 0.0001, post hoc ***P < 0.0001, n = 5, 5, 5, 5). Chronic unpredictable mild stress-induced increasing of caspase-1 activity was impeded by the hydrogen-rich water as shown in the comparison between hydrogen-rich water + stress group and stress group (F_1, 16_ = 30.06, P < 0.0001, post hoc ***P < 0.0001, n = 5, 5, 5, 5). Representative FLICA photographs from hippocampus and prefrontal cortex were shown in (**a–d**) and (**f–i**) respectively. No significant differences observed among the control, hydrogen-rich water and hydrogen-rich water + stress groups. Data was analyzed using a two-way ANOVA followed by the Bonferroni’s multiple comparisons post hoc test. Error bars represent s.e.m.

The caspase-1 activity was expressed as a percentage to the control group. The stress group had increased hippocampus caspase-1 activity compared to the control, hydrogen-rich water and hydrogen-rich water + stress groups, which indicated activation of the inflammasome (Fig. 3e). The prefrontal cortex of CUMS-treated mice exhibited significantly higher caspase-1 activity as well (p < 0.0001) (Fig. 3j). Hydrogen-rich water effectively attenuated the increased caspase-1 activity, which resulted in correlation with the excessive activation of inflammasome after CUMS treatment (Fig. 3). There were no significant differences among the control, hydrogen-rich water and hydrogen-rich water + stress groups.

### ROS production in hippocampus and prefrontal cortex

**Figure 4:**
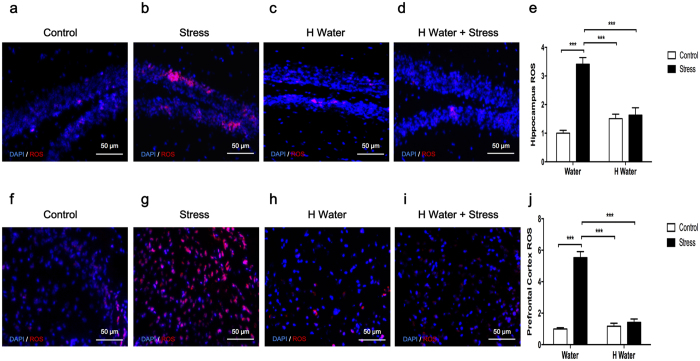
ROS production in hippocampus and prefrontal cortex. ROS production was presented as relative of the control group. (**a**) The hippocampi of chronic unpredictable mild stress exposed mice displayed over-production of ROS when compared to the control group (F_1, 16_ = 42.22, P < 0.0001, post hoc ***P < 0.0001, n = 5, 5, 5, 5), and compared to the hydrogen-rich water group (F_1, 16_ = 10.60, P = 0.0050, post hoc ***P < 0.0001, n = 5, 5, 5, 5). The stress-induced excessive production of ROS in hippocampi could be successfully inhibited by the antioxidative hydrogen-rich water as shown in the comparison between hydrogen-rich water + stress group and stress group (F_1, 16_ = 10.60, P = 0.0050, post hoc ***P < 0.0001, n = 5, 5, 5, 5). (**b**) Prefrontal cortexes of chronic unpredictable mild stress exposed mice displayed over-production of ROS when compared to the control group (F_1, 16_ = 96.18, P < 0.0001, post hoc ***P < 0.0001, n = 5, 5, 5, 5), and compared to hydrogen-rich water group (F_1, 16_ = 65.54, P < 0.0001, post hoc ***P < 0.0001, n = 5, 5, 5, 5). The stress-induced excessive production of ROS in prefrontal cortexes could be successfully inhibited by the antioxidative hydrogen-rich water as shown in the comparison between hydrogen-rich water + stress group and stress group (F_1, 16_ = 65.54, P < 0.0001, post hoc ***P < 0.0001, n = 5, 5, 5, 5). Representative ROS photographs from hippocampus and prefrontal cortex were shown in (**a–d**) and (**f–i**) respectively. No significant differences observed among the control, hydrogen-rich water and hydrogen-rich water + stress groups. Data was analyzed using a two-way ANOVA followed by the Bonferroni’s multiple comparisons post hoc test. Error bars represent s.e.m.

The ROS production was presented as relative of the control group. CUMS-exposed mice all displayed over-production of ROS in the hippocampus (Fig. 4e) and prefrontal cortex (Fig. 4j) compared to the control (p < 0.0001) and hydrogen-rich water groups (p < 0.0001). Stress-induced over production of ROS in mice brain was also successfully prevented by the antioxidative hydrogen-rich water as shown in the comparison between hydrogen-rich water + stress group and stress group (p < 0.0001) (Fig. 4e,j). No significant changes were observed among the control, hydrogen-rich water and hydrogen-rich water + stress groups.

## Discussion

Bakunina and colleagues previously demonstrated that impaired redox homeostasis result in increased oxidative stress, which can synergistically induce damaging neuroinflammation and contribute to the pathogenesis of depression^14^. This mechanism can be exacerbated by chronic stress. Che and colleagues demonstrated that CUMS exposure impairs the endogenous antioxidant defense systems and results in lipid peroxidation-induced neuronal damage and depressive-like behaviors due to accumulation of ROS^22^.

Drinking hydrogen-rich water has been shown to have protective effects against age-related oxidative damage^20^. Drinking hydrogen-rich water can reduce oxidative stress in the brain following chronic physical restraint. Moreover, studies conducted by Nagata and colleagues suggest that drinking hydrogen-rich water may be beneficial for the prevention of neuronal disorders^23^. In this study, we hypothesize that hydrogen-rich water prevents the development of depressive-like behavior. CUMS-exposed mice gained less weight relative to other three groups and displayed depressive-like behaviors, such as lower sucrose preference percentages and longer immobility times. Hydrogen-rich water significantly attenuated the depressive-like behavior exhibited by the stress group mice (Fig. 1) suggesting that hydrogen-rich water may prevent the development and progression of depressive-like behavior.

We further investigated the molecular mechanisms by which hydrogen-rich water exerted its beneficial effects on the process of depressive-like behavior. Chronic stress induced overproduction of proinflammatory cytokines (IL-6, TNF and IL-1) in the CNS, then in turn led to neuroinflammation, which played a role in the pathophysiology of depressive symptoms^3^. IL-1β mRNA and protein levels has previously been shown to remarkably increase in prefrontal cortex of depressive rats after 12 weeks CUMS procedure^24^. Our current study provides supportive evidence, demonstrating that mice exposed to 4 weeks CUMS display significant elevations in IL-1β protein expression in hippocampus and cortex. Interestingly, hydrogen-rich water could effectively prevent stress-induced elevations in CNS IL-1β protein expression (Fig. 2), which is consistent with a previous report that pretreatment with hydrogen-rich water could reduce serum IL-1β levels^18^.

The inflammasome-mediated activation of caspase-1 leads to secretion of IL-1β^25^. Suppression of IL-1β protein levels by hydrogen-rich water may be due to its inhibitory effects on inflammasome activation. We used FLICA™ reagent FAM-YVAD-FMK, a fluorescent probe that irreversibly binds to active caspase-1, for the determination of inflammasome activation^26^. The hippocampus and prefrontal cortex of CUMS-treated mice exhibited remarkable activation of capase-1, which was in agreement with our previous results and other researchers’ reports^24^,^27^. Our study also shows that stress-induced activation of caspase-1 in CNS is successfully suppressed by drinking hydrogen-rich water (Fig. 3). These data indicate that hydrogen-rich water can inhibit the activation of inflammasome.

Three integrated signals including potassium efflux, ROS over-production and lysosome destabilization are common to nearly all activators of NLRP3 inflammasome^28^. ROS production has been suggested as the more common upstream mediator. ROS inhibitors could interfere with the priming step that leads to NLRP3 inflammasome expression, and indirectly block NLRP3 inflammasome activation^29^. During mitochondrial dysfunction the NLRP3 inflammasome could be activated by mitochondrial ROS^30^. This may have clinical relevance since increased mitochondrial superoxide production is often detected in peripheral blood mononuclear cells taken from MDD patients^31^.

Therefore, we speculate that hydrogen-rich water might interfere with ROS production in the CNS as well. ROS production in the hippocampus and prefrontal cortex of stress group mice was significantly increased after 4 weeks of CUMS treatment. Notably, our study found that hydrogen-rich water suppressed ROS production in the hippocampus and prefrontal cortex of hydrogen-rich water + stress group mice (Fig. 4).

As a whole, our data indicates that stress-induced production of ROS in the CNS plays an important role in the pathophysiology of MDD. Stress-induced inflammasome activation led to increase IL-1β levels in the hippocampus and cortex. Neuroinflammation caused by IL-1β and accumulation of ROS may be key contributors to neuronal damage in the CNS. Our data further supports the notion that the inflammasome pathway may be a good target in MDD pathogenesis. Moreover, this study indicates that antioxidants have beneficial effects in the prevention of MDD. Lastly, hydrogen-rich water may be used as a novel, effective and preventative intervention of MDD if our results are further verified by findings from depressed patients in the future.

## Methods

### Animals

Eight-week-old male BALB/c mice bought from the animal center of Second Military Medical University (Shanghai, China) were entrained to a 12-h light-dark cycle in a 22 ± 2 °C animal room with food and water ad libitum, unless specified otherwise. The Animal Care Committee of Second Military Medical University approved the experiment protocol. The entire procedure was carried out in compliance with related guidelines and regulations.

### Experiment groups

Mice adapted to the new environment and a 1% sucrose solution (weight/volume) for 2 weeks before they were randomly divided into four groups. The ‘RAND ()’ function was used to generate a random number list in the Microsoft Excel 2010 software for the random divide of mice (Microsoft Corporation, St Redmond, WA, USA). The control group and stress group were supplied with tap water, while the hydrogen-rich water group and hydrogen-rich water + stress group were supplied with hydrogen-rich water. We supplied the mice with 30 ml of tap water or hydrogen-rich water according to their groups twice per day for 4 weeks at the commencement of the CUMS procedure. The average consumption volume of tap water or hydrogen-rich water per mouse was 4 ml per day. The hydrogen-rich water (0.8 ppm H_2_) was bought from Beijing Huoliqingyuan Beverage Co., Ltd. (Beijing, China).

### CUMS protocol

The CUMS protocol used in this experiment was described in our laboratory’s previous study^32^. Stressors were administered to stress group and hydrogen-rich water + stress group mice for 4 weeks in a random order. The stimuli included 45° cage tilt, cage shaking, 4 °C swimming, 45 °C oven, restraint, damp bedding, continuous illumination, and food and water deprivation.

### Sucrose preference test

This test was performed as we described in our previous paper^33^. Tap water and a 1% sucrose solution were presented to animals for 1 hour during the dark cycle. Sucrose preference was defined to be the percentage of sucrose consumption out of the total water consumption^34^. Two experimenters who were blind to the treatment carried out this test.

### Tail suspension test

We performed this test after the sucrose preference test at the end of the CUMS procedure as we described before^27^. The immobility time of each mouse during the 5 minute-long suspension was measured and analyzed by a tail suspension system (Med Associates, Inc., St. Albans, VT, USA). Another experimenter who was blind to the treatment carried out this test.

### Sample collection

The next morning following the end of 4 weeks-long CUMS procedure, all animals were sacrificed under general anesthesia with pentobarbital. The hippocampus and cortex were dissected immediately after decapitation, flash frozen in liquid nitrogen, and stored at −80 °C for further analysis.

### Enzyme-linked immunosorbent assay

The frozen hippocampus and cortex were homogenized in an ice-cold radio immunoprecipitation assay (RIPA) buffer containing 1 mM phenylmethanesulfonyl fluoride (PMSF) protease inhibitor. We collected the supernatants after the tissue lysate was centrifuged at 4 °C for 15 minutes (10,000g). Total protein concentration of supernatant was determined with a BCA assay kit. The RIPA buffer, PMSF protease inhibitor and BCA assay kit were bought from Beyotime Institute of Biotechnology (Haimen, Jiangsu, China). The concentration of IL-1β in supernatant was measured with an enzyme-linked immunosorbent assay kit (Catalog No. EM001) bought from Shanghai ExCell Biology, Inc. (Shanghai, China). We performed this assay according to the manufacturer’s established protocol. The final IL-1β levels were normalized to the total protein concentration of each supernatant accordingly.

### Measurement of caspase-1 activity

FAM-FLICA™ Caspase-1 Assay Kit (Catalog No. 97) was used to detect active caspase-1 (ImmunoChemistry Technologies, LLC. Bloomington, MN, USA). The FLICA™ reagent FAM-YVAD-FMK, a fluorescent-labeled inhibitor specific for caspase-1, could penetrate cells and covalently couple to active caspase-1 enzyme^35^,^36^,^37^. Cells retaining the bound FAM-YVAD-FMK FLICA reagent could be analyzed by fluorescence microscopy. The green fluorescent signal was a direct measure of the active caspase-1 enzyme activity.

We performed this assay according to the manufacturer-provided manual for FAM-FLICA caspase assay kits. Unfixed frozen 10 μm brain sections were prepared with Research Cryostat Leica CM3050 S (Leica Biosystems, Nussloch, Germany). The slides were allowed to air-dry and then fixed with acetone for 1 minute. After washing slides in PBS-tween twice for 15 minutes, the slides were blocked with 3% BSA in PBS-tween. The tissue section staining solution containing FLICA™ reagent was added to slides, and then incubated at 37 °C for 2 hours in a dark environment. After washing slides twice for 15 minutes, cell nuclei were stained with DAPI. Slides were imaged using an Axio Observer inverted microscope (Carl Zeiss MicroImaging, GmbH. Gottingen, Germany). The open-source image processing and analysis software ImageJ was used for the quantification of green FLICA signal and blue nuclei counting. The amount of green FLICA signal in each image was normalized to the number of nuclei accordingly.

### Detection of ROS production

Frozen 10 μm brain sections were prepared with Cryotome™ E Cryostat (Thermo Fisher Scientific, Inc., Waltham, MA, USA). Brain slides were incubated with 2.5 mM dihydroethidium (Product No. D7008) at 37 °C for 30 minutes in a dark environment (Sigma-Aldrich Co., LLC. St. Louis, MO, USA). Dihydroethidium was oxidized by ROS to ethidium bromide, which then produced red fluorescence^38^. Cell nuclei were stained with DAPI (Beyotime Institute of Biotechnology). Stained slides were examined and imaged using a Nikon Eclipse TI-SR microscope (Nikon Corporation, Shinagawa, Tokyo, Japan). ImageJ was used for quantification of the red emission signal and blue nuclei counting. The amount of red emission signal was normalized to the number of nuclei accordingly.

### Statistics

Data was presented as mean ± standard error of measurements (s.e.m.). The Shapiro-Wilk test was performed with SPSS 17.0 for Windows to confirm that all of the data sets used conformed to a Gaussian distribution profile (SPSS Science, Chicago, IL, USA). Bartlett’s test for equal variances was performed before comparing data sets. Data was analyzed using a two-way analysis of variance (ANOVA) followed by the Bonferroni’s multiple comparisons post hoc test with GraphPad Prism 6 for Mac (GraphPad Software, Inc., La Jolla, CA, USA). P < 0.05 was considered statistically significant.

## Additional Information

**How to cite this article**: Zhang, Y. *et al.* Effects of hydrogen-rich water on depressive-like behavior in mice. *Sci. Rep.* **6**, 23742; doi: 10.1038/srep23742 (2016).
